# Radiotracers for the Central Serotoninergic System

**DOI:** 10.3390/ph15050571

**Published:** 2022-05-03

**Authors:** Reynald Mangeant, Emmanuelle Dubost, Thomas Cailly, Valérie Collot

**Affiliations:** 1 Centre d’Etudes et de Recherche sur le Médicament de Normandie (CERMN), UNICAEN, Normandie Univ., 14000 Caen, France; reynald.mangeant@univ-nantes.fr (R.M.); emmanuelle.dubost@unicaen.fr (E.D.); 2 Institut Blood and Brain @ Caen Normandie (BB@C), Boulevard Henri Becquerel, 14000 Caen, France; 3 UNICAEN, IMOGERE, Normandie Univ., 14000 Caen, France; 4 CHU Côte de Nacre, Department of Nuclear Medicine, 14000 Caen, France

**Keywords:** serotonin, GPCR, radiotracer, PET, SPECT

## Abstract

This review lists the most important radiotracers described so far for imaging the central serotoninergic system. Single-photon emission computed tomography and positron emission tomography radiotracers are reviewed and critically discussed for each receptor.

## 1. Introduction

In recent decades, the serotoninergic system constituting serotonin (5-HT) and its receptors (5-HTR) has become a promising target in neuroscience. The many studies carried out have shown its involvement in many brain functions and neuropsychiatric disorders such as Alzheimer’s disease, Parkinson’s disease, schizophrenia, or depression [1].

Among the G protein-coupled receptors (GPCR), 5-HTRs appear to be the largest family of receptors. Indeed, the physiological action of serotonin is carried by at least 14 subtypes of receptors: 5-HT_1A,B,D,E,F_, 5-HT_2A-C_, 5-HT_3_, 5-HT_4_, 5-HT_5A_, 5-HT_6_, and 5-HT_7_ [2,3]. This classification (IUPHAR) is based on their signal transduction pathways, pharmacological effects, and molecular structures.

The significant variability of these receptors has led to the development of new tools, allowing the selective in vivo exploration of each of them. This exploration is based in particular on the use of medical imaging techniques such as single-photon emission computed tomography (SPECT) and positron emission tomography (PET). These non-invasive imaging techniques are commonly used by researchers and clinicians to perform the mapping of neurotransmitter systems in the living brain in healthy and sick patients. Thanks to the development and use of new radiolabeled molecules (radiotracers or radiopharmaceuticals), the precise quantification of the density of these receptors within different brain areas is possible. Radiotracers, which are molecular scaffold containing a radioactive isotope, must fulfill several criteria to be effective brain receptor imaging agents: a high selectivity and high affinity (nM range) for the target receptor, a low or moderate lipophilicity (logD = 1–3) for blood–brain barrier penetration and to avoid excessive nonspecific binding to brain tissue, a slow metabolism (or non-interfering radioactive metabolites), and a high specific activity to visualize weakly abundant biological targets.

In this review, for each 5-HTR subtype, we will focus on the central radiotracers of interest mentioned in the literature, sorted by chemical families. Their interesting properties will be discussed, and their uses in clinical or preclinical trials will be specified. Moreover, the radiotracers used for the detection and quantification of 5-HT reuptake transporters (SERTs) will be described as well. The SERT does not belong to the 5-HTR family, but its involvement in the regulation of the serotonin concentration in the inter-synaptic space makes it an important target in neuroscience and therapeutics [4].

## 2. 5-HT_1_ Receptors

5-HT_1_R receptors are involved in many biological and physiological processes and constitute a large family of receptors. Five different isoforms (5-HT_1A_, 5-HT_1B_, 5HT_1D_, 5-HT_1E_, and 5-HT_1F_) exist, all coupled to a Gαi/o protein which inhibits cAMP synthesis and therefore potentially inhibits signal transduction. In the current classification, we can note the absence of 5-HT_1C_ because the latter has been reclassified as 5-HT_2c_ following numerous discoveries for GPCRs and their sequence homology with the 5-HT_2_R family [5]. To date, the description of efficient radiotracers of the 5-HT_1_R family have been restricted to the 5-HT_1A_R and 5-HT_1B_R subtypes.

### 2.1. 5-HT_1A_ Receptors

Among the 5-HTRs, 5-HT_1A_R is the first to have been cloned and characterized. In 1987, its gene (G-21) was cloned and identified as a GPCR by structural homology with the β2-adrenergic receptor [6]. However, this is the work of Fargin et al., which determined that G-21 coded for 5-HT_1A_R [7]. In the mammalian brain, 5-HT_1A_Rs are divided into two distinct populations based on their location, including (1) presynaptic 5-HT_1A_R (autoreceptors) located in the soma and dendrites of serotoninergic neurons in the raphe nucleus [8,9], where their activation leads to a reduction in nerve impulses (Gαi/o), resulting in a decrease in the release of 5-HT in the inter-synaptic space [10,11], and (2) postsynaptic 5-HT_1A_R (heteroreceptors) localized in non-serotonergic neurons, mainly in limbic areas such as on the dendrites and soma of glutamatergic neurons, the terminal axons of GABAergic neurons, or on cholinergic neurons [12]. Their action is inhibitory and extends to the many areas of the brain innervated by the serotoninergic system. It has to be noted that 5-HT_1A_R heteroreceptors are expressed in a high density in the hippocampus (zone CA1, CA2, and serrated gyrus), the septum pellucidum, layers II and VI of the frontal cortex, the lateral and medial septum, and more moderately in the amygdala, the inferior colliculus, and in the thalamic and hypothalamic nuclei [8]. The wide distribution of 5-HT_1A_R suggests that they have a great variety of functions in the brain. Indeed, they are distinguished by their influence in the phenomena of neurogenesis, neuroprotection, neuronal plasticity, memorization, and learning, but also by their involvement in various behavioral disorders such as anxiety and depression [13,14,15]. They are currently a target of choice in medical therapy, research, and medical imaging via the development of antagonists, agonists, and radiotracers specific to this target.

Many 5-HT_1A_ receptor (5-HT_1A_R) radiotracers are described in the literature, but very few of them are currently used in human studies [16]. Indeed, there is a significant bias between the in vitro results and the different models (rodent, non-rodent, monkey, etc.) used in pre-clinical trials due to a difference in the distribution of 5-HT_1A_R in the cerebral areas of the species studied, the intensity of the signal collected compared with the background noise, and the metabolization or distribution phenomena limiting the contact between the radiotracer and its target.

### 2.2. N-Acetamide Pyridine Series: **WAY-100635** Derivatives

**Figure 1:**
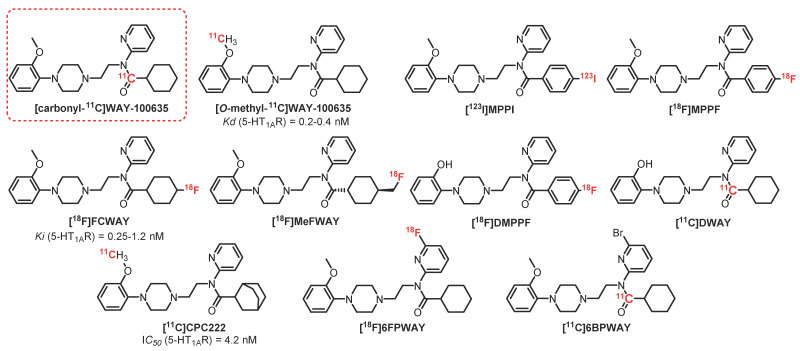
**WAY-100365** derivatives.

**[^11^C]WAY-100635** (Figure 1) is the first radioligand described as a potent antagonist, presenting good affinity for 5-HT_1A_R (K_D_ = 0.2–0.4 nM) [17]. This radioligand has shown a partial selectivity, albeit it is also known as a dopamine D4 receptor agonist (K_D_ = 2.4 nM). This lack of selectivity is not really a problem because the 5-HT_1A_R receptor density in the brain is significantly greater than the population of D4 receptors. Studies carried out on this radiotracer have shown good correlation between the distribution of receptors compared to that obtained previously using agonists and antagonists on human tissues (postmortem) [18]. The **[*O*-methyl-^11^C]WAY-100635** analogous has been the first radioligand to be synthesized for PET purposes, which led to 5-HT_1A_R images in rodents, monkeys, and humans [19,20]. However, a high level of nonspecific radioactivity has been detected in the brain due to the easy crossing of BBB by radiometabolites. Then, **[carbonyl-^11^C]WAY-100635** was synthesized, which was degraded into polar radioactive metabolites that did not cross the BBB [21,22]. Since its first synthesis, **[carbonyl-^11^C]WAY-100635** has become a reference radioligand, allowing several studies to be performed first on healthy patients and then on patients presenting several psychiatric conditions such as depression, anxiety, or anorexia [22,23,24,25]. Nevertheless, several pharmacomodulations have been performed in order to mainly reduce its metabolization, leading to the preparation of carbon-11 and fluorine-18 radioligands.

**[^123^I]MPPI**, an iodinated analogous of **[^11^C]WAY-100635**, was prepared in 1994 by Kung [26]. Nevertheless, due to the instability of the C-I bond in vivo, this compound was radiolabeled with fluorine-18, as the C-F bond is known to be less metabolized. Then, in 1997, the same group prepared **[^18^F]MPPF**, which has been proven to be a valuable 5-HT_1A_R radiotracer in monkeys [27]. **[^18^F]MPPF** is a selective antagonist of 5-HT_1A_R used to measure the evolution of serotonin levels in vivo [28]. Studies realized in rodents, cats, monkeys, and dogs led to a selective labeling of 5-HT_1A_R-rich cerebral regions (hippocampus, entorhinal cortex, and raphe nuclei) [29,30]. **[^18^F]MPPF** has also been tested on healthy patients [31] as well as patients presenting Alzheimer’s disease [32], depression, and temporal lobe epilepsy [33,34].

**[^18^F]FCWAY** is a fluorinated analogue of **WAY-100635** developed by Lang [35]. Its *trans* isomer, **[4-*trans*-^18^F]FCWAY**, exhibits a 0.25 nM affinity toward 5-HT_1A_R, and its *cis* isomer, **[4-*cis*-^18^F]FCWAY**, has a slightly weaker affinity of 1.2 nM. However, **[4-*trans*-^18^F]FCWAY** showed the best pharmacological and pharmacokinetic properties, leading to the measurement of the 5-HT_1A_R density in the hippocampus and cerebellum. **[^18^F]FCWAY** has also been used to quantify 5-HT_1A_R in patients with behavioral disorders such as epilepsy, post-traumatic stress, or panic attacks [36,37,38]. However, the accumulation of radiofluorine in bones due to the metabolism of the radiotracer decreases the resolution of brain imaging. The use of disulfiram, a cytochrome P450 isozyme 2E1 inhibitor which decreases defluorination, limited this phenomenon and improved the visualization of these receptors, but it was not sufficient to justify its use or its marketing [39].

**[^18^F]MeFWAY** is a derivative of [**^18^F]FCWAY**, possessing a fluoromethyl group at cyclohexane’s position 4, thus limiting metabolization issues. In vivo assays in rodents and primates showed that the **[*trans*-^18^F]MeFWAY** isomer presented good affinity and specificity for 5-HT_1A_R and was suitable for in vivo imaging [40,41]. In 2014, the first assays on six healthy volunteers allowed obtaining preliminary evidences of its potential as a radiopharmaceutical [42]. In 2015, Choi et al. confirmed this conclusion by using a comparative study with **[^18^F]FCWAY** associated with disulfirame [43]. Nevertheless, to date, there is no publication demonstrating its usefulness in sick patients.

**[^18^F]DMPPF** is the demethylated analogue of **[^18^F]MPPF**. This pharmacomodulation was performed in order to increase the PET signal by improving the cerebral uptake of the compound. **[^18^F]DMPPF** was crossing the BBB and leading to an efficient imaging contrast with a lower clearance in rats than **[^18^F]MPPF** [44]. Despite this encouraging first result, this radiotracer has not been used, probably due to the great interest of the community in **[^11^C]WAY-100635**.

**[^11^C]DWAY** is a putative metabolite of **[^11^C]WAY-100635** which presents similar characteristics but provides a twofold higher brain uptake than **[^11^C]WAY-100635** in the human brain [45]. However, despite these interesting results, this radiotracer has not been investigated so far.

**[^11^C]CPC-222** is a **WAY-100635** analogue with good 5-HT_1A_R affinity (IC_50_ = 4.2 nM) which crosses the BBB [46]. However, this molecule has not been used because of its too weak signal-to-noise ratio.

**[^18^F]6FPWAY** and **[^11^C]6BPWAY** are halogenated (fluorinated and brominated, respectively) analogues of **WAY-100635** [47]. Halogen atoms have been introduced on this scaffold in order to decrease the metabolism of the compound while allowing further radiolabeling with fluorine-18 or bromine-76. **[^18^F]6FPWAY** was initially synthesized and tested as [**^11^C]6FPWAY** in monkeys and then was prepared while incorporating fluorine-18 [48]. Assays have shown a moderate interest in these ligands due to faster metabolization of the compound compared with **[*O*-methyl-^11^C]WAY-100635**. This phenomenon is probably due to faster degradation of the amide bond of the 6-fluoropyridine group [49]. **[^18^F]6FPWAY** and **[^11^C]6BPWAY** have not been further studied so far.

### 2.3. 1,2,4-Triazine-3,5-Dione Series: **CUMI-101** Derivatives

**Figure 2:**
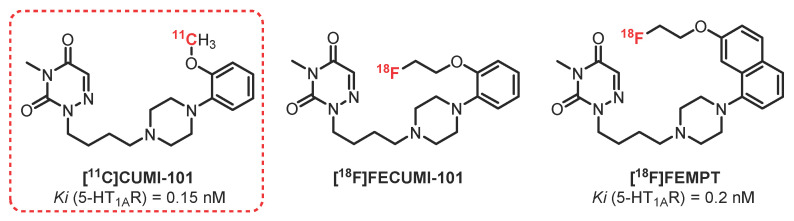
**CUMI-101** derivatives.

**[^11^C]CUMI-101** and its derivative **[^11^C]FECUMI-101** are 5-HT_1A_R agonists (Figure 2). Agonists are good candidates for estimating the level of 5-HT receptors present in the synaptic cleft, the receptor’s occupancy, and the determination of the internalization of autoreceptors after administration of the serotonin transporter’s (SERT) inhibitors, which are used to treat depressive states. **[^11^C]CUMI-101**, also called **[^11^C]MMP**, is a 2-methoxyphenyl analogous of a previous radiotracer (**MPT**) described by the same authors in 2006. This former radiotracer has shown a promising agonist profile with good affinity for 5-HT_1A_R (Ki = 1.36 nM) in baboons [50], but slow washout kinetics prompted the authors to perform structure–activity relationship studies, leading to the preparation of **[^11^C]CUMI-101**. This compound has been demonstrated to be a potent (Ki = 0.15 nM) and selective 5-HT_1A_R agonist ligand, with a high uptake in 5-HT_1A_R-rich areas in baboons [51]. Used in healthy volunteers, **[^11^C]CUMI-101** led to determination of the distribution and quantification of central and peripheral 5-HT_1A_R [52,53]. Notably, this radiotracer shows interesting properties, as its metabolites are not crossing the BBB. In 2011, Hendry et al. investigated the pharmalogical profile determination of **[^11^C]CUMI-101** in rat brain tissue and demonstrated that, while acting as an 5-HT_1A_R agonist in humans, it behaves as an antagonist in the rat cortex and rat hippocampal tissue [54]. Thus, this compound may act as a partial agonist, leading to interpretation difficulties from one model to another. In 2020, **[^11^C]CUMI** was also used to assess the 5-HT_1A_R occupancies of brexpiprazole in adult subjects with schizophrenia [55].

**[^18^F]FECUMI-101** is the ethylfluorinated analogue of **[^11^C] CUMI-101** synthesized in 2013 by Kumar [56]. Studies carried out in baboons showed that this radiotracer crosses the BBB with good distribution of the radiotracer in the 5-HT_1A_R-rich brain regions, with the exception of the thalamus. In 2016, autoradiographic studies carried out on human brain sections confirmed this specific binding, along with some binding with α1 receptors [57]. A possible explanation is the difference in the concentration of α1 receptors in different species in this brain region or a specific binding on an unknown target. In addition, competition experiments in the presence of **WAY-100635** have shown a binding blockade of **[^18^F]FECUMI-101** on the receptors present in the cerebellum, limiting its use as a reference for in vivo PET applications.

**[^18^F]FEMPT** is a radiotracer synthesized in 2017 by Collier et al. via a continuous flow microfluidic method [58]. The advantage of this technique lies mainly in the saving of time and the reagent, allowing one to increase and optimize the parameters and the yield of reactions for radiolabeling with fluorine-18. The first in vitro assays carried out with this compound showed a strong affinity of **[^18^F]FEMPT** toward 5-HT_1A_R (Ki = 0.2 nM). In addition, the affinity for α1 adrenergic receptors (Ki = 180 nM) was much lower than that observed for **[^11^C]CUMI-101**. These initial results are very encouraging, and preclinical studies are currently being carried out on this new radiotracer.

### 2.4. 2-Pyridinemethylamine Series: **F15599** Derivatives

**Figure 3:**
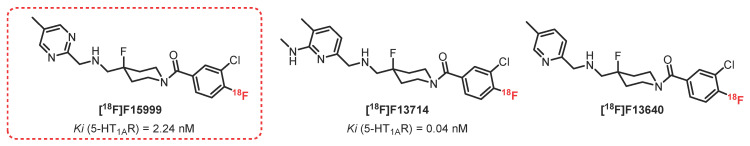
**F15999** derivatives.

**F15599** (Figure 3) is the first 5-HT_1A_R ligand described in the 2-pyrimidinemethylamine series, presenting a strong affinity and very high specificity for 5-HT_1A_R. In vitro and in vivo studies led to assessing its particular affinity for the postsynaptic 5-HT_1A_R present in the cortical areas of the brain [44]. In vivo imaging with **[^18^F]F15599** in rats and cats demonstrated a rapid accumulation of this radioligand in the brain, suggesting good BBB passage as well as good distribution in brain areas such as the raphe nucleus, the hippocampus, and the side bridge [45]. However, the labeling intensity was considered insufficient for further investigations as a radiopharmaceutical, probably due to its relatively low affinity (Ki = 2.24 nM) [16]. Efforts have been therefore focused on pharmacomodulations of **[^18^F]F15599**.

**[^18^F]F13714**, a 2-pyridinemethylamine analogue of **[^18^F]F15999**, presented a particularly strong affinity for recombinant human 5-HT_1A_R (Ki = 0.04 nM) [59,60]. In vitro autoradiography carried out on rats revealed significant radioactivity in the targeted brain areas [61]. In addition, **[^18^F]F13714** was shown to have very good resistance to metabolism. Specific binding of this radiotracer in 5-HT_1A_R-dense areas was observed in in vivo PET imaging (cats), with a particular tropism for the cortex compared with the hippocampus. In 2016, Yokoyama et al. published a comparative study between **[^18^F]F13714** and **[^18^F]MPPF** in awake and anesthetized marmosets, demonstrating the strong influence of anesthesia on functional 5-HT_1A_R and therefore suggesting that caution is necessary when interpreting results from agonists in PET imaging [62]. Although the results were encouraging, **[^18^F]F13714** was not optimal for in vivo imaging because of its irreversible binding.

**F13640** (also known as **befiradol** or **NLX-112**) is a 5-HT_1A_R ligand that has recently been investigated from the same series, presenting selectivity and efficacy for 5-HT_1A_R along with strong analgesic properties [63]. Demonstrated on a large panel of animal models presenting physiological or pathological acute pain, and in comparison with a large intake of opioids, its analgesic action led to the development of a reverse tolerance [64]. In 2018, Zimmer et al. carried out the first in vivo imaging assays in rats, cats, and primates [65]. **[^18^F]F13640** has been proven to cross the BBB (without formation of radioactive metabolites), and its distribution has been observed in 5-HT_1A_R-rich areas, with notable differences in comparison with the distribution observed using antagonist radiotracers so far. In addition, **[^18^F]F13640** has been used to determine the evolution of the number of these receptors in the hippocampus in the prodromal stage of Alzheimer’s disease (post mortem) [66]. In 2019, **[^18^F]F13640** was used in vivo in healthy humans, affording promising results since the visualized distribution correlated with that of functional 5-HT_1A_R receptors [67]. **[^18^F]F13640** is the first radiopharmaceutical that enables in vivo investigation of functional 5-HT_1A_R that is likely to be altered in pathological conditions such as neuro-degenerative diseases or psychiatric disorders. Indeed, in 2021, **[^18^F]F13640** was proven to be a valuable tool to explore neurological or neuropsychiatric pathologies involving fluctuations in extracellular serotonin. Indeed, Zimmer et al. have shown its sensitivity to detecting in vivo 5-HT concentration fluctuations [68].

### 2.5. 5-HT_1B_ Receptors

Like 5-HT_1A_R, 5-HT_1B_R exists at the pre- and postsynaptic level. However, their locations are different; presynaptic 5-HT_1A_R is localized mainly in the cell body and the dendrites of the serotoninergic neurons, while 5-HT_1B_R is expressed at the axonal endings. The 5-HT_1B_R has an important influence in the regulation of the concentration of 5-HT in the intersynaptic space and induces a negative feedback limiting 5-HT’s release (homoreceptors). All postsynaptic 5-HT_1B_R (heteroreceptors) receptors are located on the terminal axons of non-serotonergic neurons (GABAergic, muscarinic, or dopaminergic) and will influence the release of their neurotransmitters. There is a great homology between the receptors present in humans and rats, with the only difference being a replacement of a threonine by an asparagine on the seventh transmembrane domain. However, this slight difference is responsible for significant variations during pharmacological studies. The 5-HT_1B_R receptors are present in a high density in different brain locations, such as the globus pallidus, substance nigra, ventral pallidum, or dorsal subiculum and more moderately in the cortex, the hippocampus, and the putamen [69]. The 5-HT_1B_R receptors are involved in learning and memorization phenomena. Thus, the injection of antagonists into knockout mice allowed the observation of an improvement in their cognitive performance, with a probable involvement of the cholinergic system [70]. In addition, pharmacological studies have demonstrated the interest of 5-HT_1B_R in aggression phenomena, with a decrease of the latter in the presence of agonists [71].

### 2.6. Chromen-4-One Series: **AZ10419369** Derivatives

**Figure 4:**
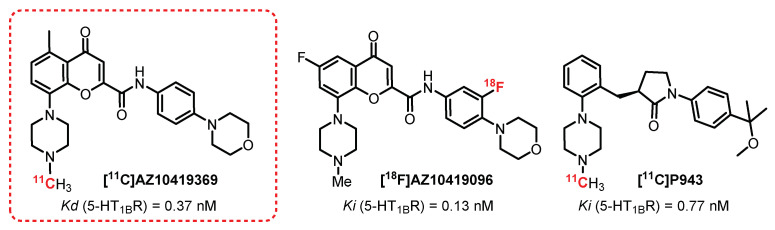
**AZ10419369** derivatives.

**[^11^C]AZ10419369** (Figure 4) is a partial 5-HT_1B_R agonist (K_D_ = 0.37 nM). This compound is considered a reference radiotracer for these receptors and has been used on numerous occasions to assess the influence of 5-HT_1B_R in healthy volunteers as well as in neuropsychiatric disorders. In 2008, Pierson et al. carried out the first studies in macaques and observed a specific radiolabeling of 5-HT_1B_R-rich areas through a competitive method in the presence of a reference antagonist: **AR-A000002** [72]. In addition, the interesting potential of this compound has been confirmed thanks to the intensity of the collected signal and the absence of detection of any radiometabolite. In this same study, **[^11^C]AZ10419369** was tested in two human subjects, and good correlation in the receptor’s distribution in both macaques and human brains was observed, as well as those obtained previously by autoradiogram in guinea pig brains [73]. From 2010 to 2020, many studies used **[^11^C]AZ10419369** as a reference in PET imaging [74,75]. Used in patients with depressive disorders and Parkinson’s disease, a decrease in 5-HT_1B_R in the targeted areas of the brain was observed [76,77]. Very recently, pharmacomodulations of **[^11^C]AZ10419369** have been performed by Lindberg et al. in order to replace carbon-11 with the longer half-life fluorine-18, allowing them to increase the acquisition time in imaging [78,79]. Among the described radiotracers, **[^18^F]AZ10419096**, a radiotracer published in 2019 by Lindberg et al. [80], revealed interesting properties. This compound is an antagonist of 5-HT_1B_R presenting very good affinity (Ki = 0.13 nM). The first in vivo tests in monkeys demonstrated a specific binding of this compound to 5-HT_1B_R-rich brain areas (e.g., the occipital cortex or globus pallidus). Approximately 80% of this binding is inhibited in the presence of a reference antagonist (pretreatment with **AR-A000002**). In addition, the main metabolites of **[^18^F]AZ10419096** seemed to be too polar to cross the BBB. The only drawback of this compound lies in its synthesis, since the yield of radiolabeling from the boronic precursor is very low (<5%) and needs to be optimized.

**[^11^C]P943**, obtained through a structural simplification of **[^11^C]AZ10419369**, is a 5-HT_1B_R antagonist presenting a very strong affinity and specificity for these receptors (Ki = 0.77 nM). A complete pharmacokinetic study was performed in baboons (*Papio anubis*) in order to determine the BBB crossing, the presence of metabolites, and its affinity for 5-HT_1B_R. The specificity of **[^11^C]P943** for 5-HT_1B_R was revealed by blocking the receptors with a specific 5-HT_1B_R antagonist (**SB-616234-S**), while no significant change was observed using a 5-HT_1D_R antagonist (**SB-714786**) [81]. Thanks to this radiotracer, the implication of 5-HT_1B_R was evaluated in patients with various pathologies (e.g., depression or post-traumatic stress) or drug addiction (cocaine), where a decrease in the binding of **[^11^C]P943** to 5-HT_1B_R was observed compared with the healthy patients [82,83]. This phenomenon is reversed in people with alcohol addiction, where an increase in binding was observed, especially in the ventral striatum [84].

## 3. 5-HT_2_ Receptors

**Figure 5:**
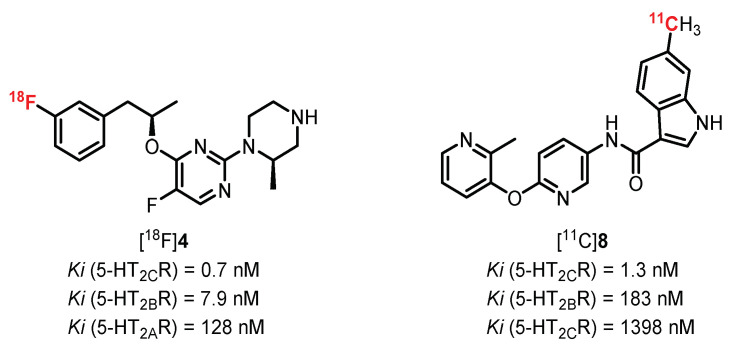
5-HT_2C_R selective radioligands.

5-HT_2_R receptors are among the first serotoninergic receptors to be identified with 5-HT_1A_R. Their identification was possible thanks to genetic cloning, allowing for distinguishing three different subtypes (5-HT_2A_, 5-HT_2B_, and 5-HT_2C_). Structural variations in these 3 isomers with 471 amino acids for 5-HT_2A_R, 481 for 5-HT_2B_R, and 458 for 5-HT_2C_R (in humans) can be noted. This variation in structure is not surprising, since each of them is encoded by different genes carried on three distinct chromosomes (chromosomes 13, 2, and X, respectively). These receptors possess a wide area of localization and are mainly involved in cell development and migration processes [85]. All these receptors are coupled to a G_q/11_ protein, and their activation regulates an enzymatic cascade involving the phospholipase C. This enzyme leads to the hydrolysis of membrane phosphatidylinositol biphosphate into two intracellular secondary messengers: diacylglycerol and inositol-1,4,5-triphosphate (IP_3_). IP_3_ plays an important role in the release of calcium ions by binding to the IP_3_ receptors on the membrane of the endoplasmic reticulum. This increase in Ca^2+^ ions in the cytosol is completed by the action of ion pumps (allowing the passage of extracellular Ca^2+^ ions) which are activated by protein kinases C, themselves activated by the diacylglycerols synthesized beforehand [86]. Currently, there are no potent and selective radioligands commonly used in clinic or research facilities to quantify and characterize 5-HT_2B_R and 5-HT_2C_R. It is nevertheless interesting to note the recent development of promising new molecules that are affine and selective for 5-HT_2C_R, specifically marking the choroid plexus (cerebral area rich in 5-HT_2C_R) (Figure 5) [87,88]. These molecules could be the subject of future clinical studies and could enhance the interest shown in these receptors via PET and SPECT imaging.

Conversely, numerous studies involving 5-HT_2A_R radioligands have been described in the literature. In humans, 5-HT_2A_R was identified and cloned for the first time by Branchek et al. in 1990 [89]. It quickly aroused the particular interest of the scientific community because of its role in certain behavioral disorders and its psychomimetic effects when taking psychotropic substances such as lysergic acid diethylamide (LSD), mescaline, or psilocybin [90]. Human 5-HT_2A_R radioligands are located mainly in the cortex and in a lower density in the central brain structures such as the hippocampus or the caudate nucleus. In the cortex, they are found mainly in layers II–III but also in lesser quantities in layers V and VI [91]. At the cellular level, they are mainly postsynaptic receptors and are expressed in non-serotonergic (cholinergic or GABAergic) neurons with the result of increasing cellular hyperexcitability and promoting nerve impulses [92]. 5-HT_2A_R has been at the center of many studies on the one hand for its involvement in many neuropsychiatric disorders such as depression, anxiety, schizophrenia, and psychosis and on the other hand because it is the target of second-generation antipsychotics (D1/D2 and 5-HT_2A_ antagonists) such as Clozapine, Olanzapine, or Risperidone [93]. To date, many efficient 5-HT_2A_R radiotracers have been developed in several chemical series both for SPECT and PET.

### 3.1. Quinazoline-2,4-Dione and Thiazolo [3,2-a]Pyrimidin-5-One Series: **Ketanserin** Derivatives

**Figure 6:**
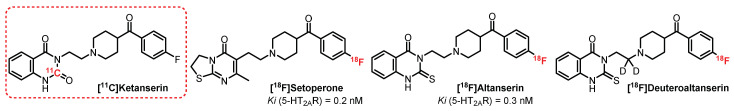
**Ketanserin** analogues as 5-HT_2A_R radiotracers.

The first evaluations of 5-HT_2_R radiotracers were carried out in 1985 using a very affine and selective antagonist (in vitro and in vivo in rats): **[^11^C]Ketanserin** [94]. In humans, a too rapid metabolization along with nonspecific binding rapidly stopped the development of this molecule as a 5-HT_2_R radiotracer. However, these first trials were the starting point for more specific studies of 5-HT_2A_R in PET imaging through the development of **ketanserin** analogues (Figure 6).

**[^18^F]Setoperone** is one of the reference radioligands for 5-HT_2A_R. It is a very potent antagonist of these receptors (Ki = 0.2 nM) along with relative specificity since in baboons, it also interacts with the dopaminergic D2 receptors present in the striatum [95]. Despite this specificity issue, it has been used regularly as a radiotracer for 5-HT_2A_R in PET imaging, especially in healthy subjects or subjects with various pathologies such as depression, migraines, or Alzheimer’s disease [96,97,98,99,100]. **[^18^F]Setoperone** is not used anymore in human studies due to the discovery of **[^18^F]Altanserin**, which has demonstrated a higher potential in animal and human studies.

As a 5-HT_2A_R antagonist (Ki = 0.3 nM), **[^18^F]Altanserin** was first evaluated in rats in 1991 by Lemaire et al., and an important signal in the frontal cortex along with a more moderate one in the striatum were detected [101]. The specificity of the signal was determined by prior blocking of the receptors in the presence of 5-HT_2A_R ligands (ketanserin, pipampone, and methylsergide) and D2 receptor ligands (sulpiride and halopemide). Subsequently, **[^18^F]Altanserin** was tested in healthy volunteers, and good distribution of the radioligand was found despite the presence of nonspecific binding, particularly in the cerebellum [102]. This off-target labeling was later attributed to the in vivo production of radiometabolites [103,104]. A more recent study on 52 healthy volunteers demonstrated the absence of a significant difference in **[^18^F]Altanserin** binding between the male and female genders and a significant decrease in 5-HT_2A_R in the elderly and in patients with high body mass indexes [105]. **[^18^F]Altanserin** has also been used to determine the density of 5-HT_2A_R in subjects with various CNS-related disorders such as anorexia, schizophrenia, obsessive-compulsive disorder, depression, and Alzheimer’s disease [105,106,107,108,109]. In light of all these characteristics, numerous pharmacomodulations of **[^18^F]Altanserin** were considered in order to increase the resolution of PET imaging by limiting nonspecific binding and by decreasing the production of lipophilic radioactive metabolites.

In 2001, in order to reduce metabolite production, a deuterated analogue was considered: **[^18^F]Deuteroaltanserin**. The main objective was to maintain the pharmacological and pharmacokinetic properties while delaying the metabolization by inserting an isotope of hydrogen: deuterium. Initially, a study was carried out in baboons and did not allow observing any significant difference between the two radioligands (neither for radiolabeling nor metabolization) [110]. Subsequently, a study on a healthy man was conducted and allowed the observation of good distribution of the radiotracer as well as a modest decrease in the production of radiometabolites in the plasma [111,112]. In 2009, Santhosh et al. carried out a study with **[^18^F]Deuteroaltanserin** on nine subjects affected by Alzheimer’s disease and concluded that there was a significant reduction in the binding of the radiotracer to 5-HT_2A_R, mainly in the anterior, frontal, medial, parietal, and temporal cingulate cortex [113]. Despite these promising results, no study has referred to **[^18^F]Deuteroaltanserin** since then.

### 3.2. Piperidin-4-Ylmethanol Series: **Volinanserin** Derivatives

**Figure 7:**
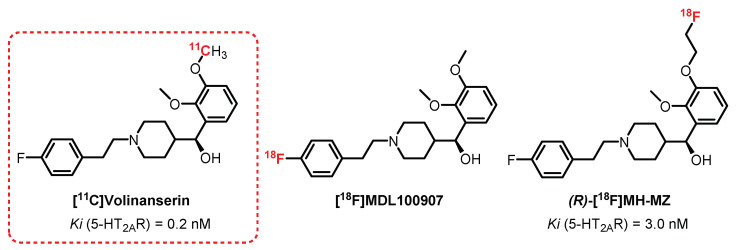
**Volinanserin** analogues as 5-HT_2A_R radiotracers.

**[^11^C]Volinanserin**, or **[^11^C]MDL100907** or **[^11^C]MDL100,907** (Figure 7), is a very selective and potent 5-HT_2A_R antagonist (Ki = 0.2 nM) [114]. Its interesting pharmacokinetic properties, such as its moderate lipophilicity (logP = 2.7), allowed **[^11^C]Volinanserin** to become a reference radiotracer in the study of the distribution of 5-HT_2A_R. First, its tritiated analogue **[^3^H]MDL10090** has been used in animals (rats and monkeys) to assess its strong specificity, allowing the visualization, quantification, and direct characterization of these receptors by autoradiography [115,116]. The monkey studies were enlarged to the use of **[^11^C]Volinanserin** in PET imaging by Lundkvist et al. [117] and permitted the observation of specific labeling of the frontal, temporal, and visual cortex through competition with **ketanserin**. In 1998, Ito et al. confirmed these results in healthy humans (three volunteers), where a specific labeling of the neocortex (occipital, frontal, and temporal cortex) with moderate nonspecific labeling was observed [114]. In 2012, a comparative study on a large cohort of healthy volunteers consolidated the interest in **[^11^C]Volinanserin** since, in addition to obtaining good distribution of labeling of 5-HT_2A_R receptors, a decline in this population with age for 5-HT_2A_R expression was evidenced. In addition, the analyses carried out demonstrated a slow metabolization of the radiotracer limiting the presence of radiometabolites, the main drawback encountered with **ketanserin** derivatives [118]. **[^11^C]Volinanserin** represents a reference ^11^C-radiotracer for the study of 5-HT_2A_R receptors and has been used in numerous studies on humans and animals in order to observe the influence of 5-HT_2A_R in various pathologies, such as depression, Huntington’s disease, Asperger’s syndrome, as well as in obsessive-compulsive disorders [119,120,121]. Interesting fluorinated analogues of **Volinanserin** have also been evaluated recently. **[^18^F]MDL100907** is a **Volinanserin** analogue labeled with fluorine-18 instead of carbon-11, and it was considered mainly for the benefit represented by the longer half-life of fluorine-18 compared with carbon-11. Few publications refer to this radiotracer, mainly because of the difficulty in achieving an efficient radiofluorination of **Volinanserin** [122]. In 2014, a study carried out by Ren et al. identified a new method for obtaining **[^18^F]MDL100907** using a nickel-mediated fluorination strategy. In nonhuman primates, a comparison of binding results between **[^18^F]MDL100907** and **[^11^C]Volinanserin** concluded that **[^18^F]MDL100907** was able to provide a better 5HT_2A_R distribution and density through PET imaging [123]. ***(R)*-[^18^F]MH-MZ** is an ethylfluorinated analogue of **[^11^C]Volinanserin** which exhibits very good affinity and specificity for 5-HT_2A_R (Ki = 0.72 nM), along with easier radiosynthetic access compared with **[^18^F]MDL100907**. In rats, the ***(R)*-[^18^F]MH-MZ** enantiomer showed the best radiolabeling results, as the affinity of the racemic mixture **[^18^F]MH-MZ** was quite lower (Ki = 3.0 nM) [124,125]. The comparative study between **[^18^F]MH-MZ** and **[^18^F]Altanserin** demonstrated a more specific labeling of areas rich in 5-HT_2A_R by **[^18^F]MH-MZ**. In addition, it revealed a low level of radiometabolites in the cortex extracts compared with the plasma levels, thus confirming that the latter cannot pass the BBB [126]. Despite these encouraging results, to date, no publication refers to the use of this radiotracer in humans.

### 3.3. N-Benzylphenethylamine Series: **CIMBI-5** Derivatives

**Figure 8:**
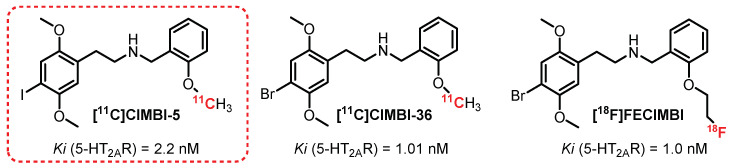
**CIMBI-5** analogues as 5-HT_2A_R radiotracers.

The vast majority of radiotracers synthesized to radiolabel 5-HT_2A_R are antagonists of these receptors. However, the development of a series of agonist compounds constitutes a major interest, allowing for distinguishing the functional receptors involved in various brain pathologies. It is in this context that **[^11^C]CIMBI-5** was synthesized (Figure 8). In 2010, Ettrup et al. carried out the first studies on this compound, which revealed that it was indeed a potent 5-HT_2A_R agonist with moderate specificity for these receptors (Ki (5-HT_2A_R) = 2.2 nM; Ki (5-HT_2B_R) = 2,3 nM; Ki (5-HT_2C_R) = 7.0 nM) [127]. The ex vivo study carried out on rat brains showed absorption of the radiotracer mainly in the frontal cortex equivalent to that obtained with **[^18^F]Altanserin**. This binding is specific to 5-HT_2A_R, as proven by a significant displacement of the radiotracer in the presence of **ketanserin**. In vivo (pigs) imaging revealed significant cortical absorption as well as more moderate absorption in the thalamic and striatal regions, thus evidencing the interest in evaluating **[^11^C]CIMBI-5** in humans. In 2019, additional tests carried out on nonhuman primates (two baboons and two vervet monkeys) allowed observing a radiolabeling in correlation with those obtained previously but with a lower intensity compared with **[^11^C]Volinanserin** [128].

Many derivatives of **[^11^C]CIMBI-5** were synthesized and tested, with widely varying radiolabeling results [129]. Among them, **[^11^C]CIMBI-36**, the brominated analogue of **[^11^C]CIMBI-5**, demonstrated the most interesting results. **[^11^C]CIMBI-36** possesses a strong affinity and specificity toward 5-HT_2A_R (Ki = 1.01 nM) and afforded an intense radiolabeling of the cerebral cortex in pigs [129]. In 2014, **[^11^C]CIMBI-36** was evaluated against a cohort of 29 healthy volunteers, and good distribution of the radiotracer was demonstrated, particularly in the neocortex and the sensory cortex. The study suggested a weak plasmatic stability of **[^11^C]CIMBI-36**, with a 50% decrease in plasma 10 min after injection. This metabolism issue was not evidenced in the pig brain tissue but was the subject of additional research [130,131]. Finally, in the presence of **ketanserin**, radiolabelling was significantly reduced in the neocortex, confirming the binding selectivity toward 5-HT_2A_R. Additional studies were carried out on humans and animals with the aim of observing the changes induced by the administration of substances that interact with the serotonergic system [132,133,134]. The study on pigs and monkeys revealed a decrease in the uptake of **[^11^C]CIMBI-36** (46%) in the presence of a significant increase in 5-HT. In humans, **[^11^C]CIMBI-36** allowed assessing a decrease in radioemission in areas rich in 5-HT_2A_R in the presence of D-amphetamine, thus evidencing the interest in this radiotracer as a potential new tool for the evaluation of neuropathologies such as depression [135]. Finally, in 2016, a study by Ettrup et al. revealed a probable binding of **[^11^C]CIMBI-36** to 5-HT_2C_R detected by radiolabeling of the hippocampus and choroid plexus greater than that in the presence of **[^18^F]Altanserin** [136].

**[^18^F]FECIMBI**, an ethylfluorinated analogue of **[^11^C]CIMBI-36**, exhibited interesting binding properties toward 5-HT_2_Rs (Ki (5-HT_2A_R) = 1 nM; Ki (5-HT_2C_R) = 1.7 nM) and an agonist profile. **[^18^F]FECIMBI** was synthesized by Prabhakaran et al. in 2015 and was able to radiolabel the temporal cortex, the hippocampus, and the choroidal plexus (an area rich in 5-HT_2C_R) by autoradiography [137]. In 2017, a complementary study in monkeys showed insufficient cerebral absorption and an insufficient volume of distribution for the development of an in vivo quantification tool [138].

### 3.4. Miscellaneous Derivatives as SPECT Imaging Tracers

**Figure 9:**
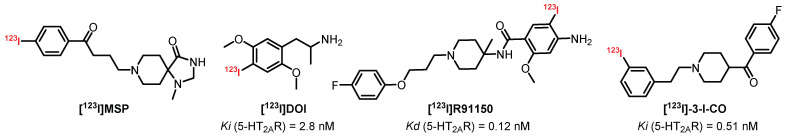
Miscellaneous 5-HT_2A_R SPECT imaging tracers.

Over the past few decades, many SPECT radiotracers with an affinity for 5-HT_2A_R have been synthesized (Figure 9). However, due to moderate selectivity, the presence of radiometabolites interfering with imaging and signals that are too weak, very few of them are currently used to quantify 5-HT_2A_R in humans. Discovered in the 1970s, **[^123^I]DOI** is a mixed agonist of 5-HT_2A_R and 5-HT_2C_R which allowed the first scintigraphic explorations of the brain even before the emergence of SPECT and PET imaging techniques [139]. **[^123^I]DOI** was previously used in its racemic form, in particular in rats (Ki = 2.8 nM) [140]. In 1989, Nazarali et al. published a comparative study highlighting the interest of ***(R)*-[^125^I]DOI** (Kd = 1.2 nM) in its enantiomer ***(S)*-[^125^I]DOI** (Kd = 2.1 nM) [141]. Finally, the results obtained for baboons did not conclude favorably with regard to its use as a specific radiotracer for 5-HT_2A_R and 5-HT_2C_R [142].

**[^123^I]R91150** or **[^123^I]R93274** are the most popular radiotracers used to quantify and characterize 5-HT_2A_R by SPECT imaging. **[^123^I]R91150** is a potent (Kd = 0.12 nM) and selective antagonist of 5-HT_2A_R which demonstrated its effectiveness through pronounced radiolabeling of the frontal cortex (FC) compared with the cerebellum (CER, nonspecific), with an FC/CER ratio greater than 10 in rats (in vivo) [143]. In 1997, Abi-Dargham et al. published the first complete preclinical study carried out on baboons and observed a specific radiolabeling of the occipital and temporal cortex (reversible in the presence of **ketanserin**) with a lower FC/CER ratio (1.5). The plasma study revealed a rapid metabolization of **[^123^I]R91150** (75% metabolized 3 h after the injection) with the presence of a less lipophilic metabolic fraction which did not cross the BBB [144]. These observations were confirmed by studies carried out on healthy volunteers who did not question the usefulness of this radiotracer for the quantification and characterization of cerebral 5-HT_2A_R [145,146]. In 1998, **[^123^I]R91150** was tested on a larger cohort of healthy volunteers (13 women and 13 men) and confirmed the results obtained with **[^18^F]Altanserin** (i.e., the absence of a significant difference between genders and the significant decrease in 5-HT_2A_R with aging) [147]. During the last two decades, **[^123^I]R91150** was used on numerous occasions to study various neuropathologies such as Alzheimer’s disease, Parkinson’s disease, or schizophrenia [148,149,150]. **[^123^I]R91150** became a reference for evaluating the distribution and the quantification of 5-HT_2A_R in subjects (humans and animals) presenting neuropsychiatric disorders (with or without treatment) such as depression, anxiety, anorexia, or having suicidal behaviors [151,152,153,154,155,156,157]. In 2009, a fluorinated analogue usable in PET imaging was synthesized: **[^18^F]R91150**. Despite encouraging results, the difficulty of obtaining the radiotracer did not encourage its development for further studies [158].

**[^123^I]MSP** is an iodinated derivative of **spiperone**, an atypical antipsychotic drug belonging to the butyrophenone family used as a treatment for schizophrenia. **[^123^I]MSP** was designed to maintain the affinity for 5-HT_2A_R of **spiperone** while decreasing its affinity toward dopaminergic D2 and D3 receptors. The study carried out with **[^123^I]MSP** evidenced a strong affinity and selectivity for 5-HT_2A_R, with a radiolabeling distribution in correlation with those obtained with the reference radiotracers (intense labeling of the mouse frontal cortex) [159]. Despite promising results, **[^123^I]MSP** has not been the subject of recent studies.

**[^123^I]-3-I-CO** is a potent (Ki = 0.51 nM) and selective antagonist of 5-HT_2A_R which was developed by Fu et al. in 2002. In 2008, a study on rodents showed good distribution of the radiotracer with a moderate intensity and poorly specific signal. In addition, **[^123^I]-3-I-CO** was the target of efflux mechanisms at the BBB level, demonstrated by a significant increase in the intensity of the radiosignal after prior administration of a P-glycoprotein inhibitor (cyclosporine A) [160]. The potential of this radioligand was therefore limited for clinical development and was simply abandoned.

## 4. 5-HT_3_ Receptors

Among the 14 serotonin receptor subtypes, 5-HT_3_R is the only ionotropic receptor which belongs to the pentameric ligand-gated ion channel (LGIC) superfamily [161]. In humans, there are five 5-HT_3_R subtypes, from 5-HT_3A_ to 5-HT_3E_. The subunits are encoded by the HTR_3_ genes located on chromosome 11q23 (HTR_3A_ and HTR_3B_) and 3q27 (HTR_3C_, HTR_3D_, and HTR_3E_) [162]. 5-HT_3_R is found in both the CNS and peripheral nervous system. It is mainly located in the dorsal vagal complex of the brainstem, regions involved in the vomiting reflex, and in low levels in many forebrain areas, including the hippocampus, amygdala, nucleus accumbens, putamen, and caudate nucleus [163,164]. The 5-HT_3_Rs are involved in many physiological processes and neuropsychiatric disorders such as schizophrenia, depression, bulimia, anxiety, pain, autism, bipolarity, and learning and memory disorders [165,166,167]. A class of high-affinity 5-HT_3_R antagonists named “setrons” has been established as antiemetic drugs, resulting from chemo- and radiotherapy and general anesthesia [166,168]. Moreover, many selective agonists and antagonists are described, thereby facilitating the development of promising radioprobes. However, to date, no radiotracers have demonstrated useful applications in humans.

### 4.1. Tropane Series: **MDL7222** Derivatives

**Figure 10:**
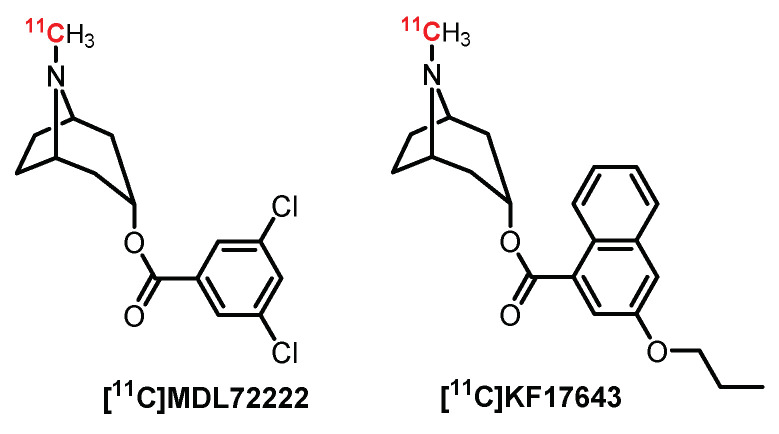
**[^11^C]MDL72222** and **[^11^C]KF17643**.

First, several antagonists labeled with carbon-11 (Figure 10) were designed as potential PET radiotracers [169]. Thus, **MDL72222**, a selective 5-HT_3_R antagonist, was radiolabeled with carbon-11 and further evaluated in rats and baboons. Despite **[^11^C]MDL72222** quickly crossing the BBB in preliminary studies, a heterogeneous diffusion in the brain was observed, probably due to its high lipophilicity and low specificity [170]. A closely related analogue of **MDL72222** was radiolabeled by Ishiwata et al. for PET imaging: **[^11^C]KF17643**. Nevertheless, **[^11^C]KF17643** displayed nonspecific binding [171].

### 4.2. Pyrrolo[1,2-a]Pyrazine Series: **S21007** Derivatives

**Figure 11:**
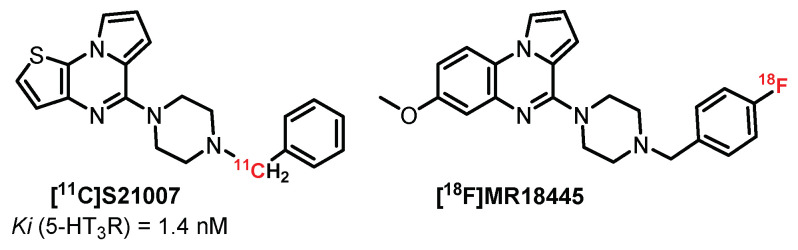
**[^11^C]MDL72222** and **[^11^C]KF17643**.

**S21007** is a 5-HT_3_R partial agonist (Figure 11) which has also been evaluated as a potential PET ligand in rats and baboons because of its strong affinity (Ki = 1.4 nM), selectivity, and high brain uptake. However, **[^11^C]S21007** was unable to detect the 5-HT_3_R via a specific binding signal, as **[^11^C]S21007** did not displace unlabeled **S21007** in the brain areas with 5-HT_3_ receptors, suggesting a lack of specific binding [172]. This series was further investigated with the synthesis of [^18^F]**MR18445**, another pyrrolo[1,2-*a*]pyrazine derivative radiolabeled with fluorine-18. Autoradiography and PET studies showed rapid uptake in both rat and baboon brains, but no specific binding in 5-HT_3_R-rich brain regions was detected with **[^18^F]MR18445,** probably due to its high lipophilicity [173]. All these compounds were not successfully used for selective binding to 5-HT_3_R in vivo.

### 4.3. (S)-Quinuclidin-3-Amine Series: **Zacopride** Derivatives

**Figure 12:**
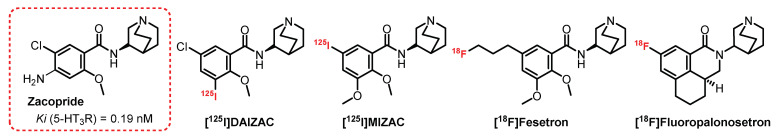
**Zacopride** derivatives as 5-HT_3_R radiotracers.

**Zacopride**, a 3-aminoquinuclidinyl derivative, is a well-known mixed 5-HT_3_R antagonist and 5-HT_4_R agonist [174]. A series of **Zacopride** derivatives was developed by Ebert et al. (Figure 12) [175,176]. **[^125^I]DAIZAC** exhibited high affinity (Ki = 0.19 nM) and enhanced selectivity for the 5-HT_3_R. Autoradiographic studies also demonstrated the usefulness of this ligand, but to date, despite these promising characteristics, the ligand has not yet been further developed for SPECT imaging [176]. Highly selective and potent antagonists, such as granisetron and palonosetron, belong to a family of drugs (the “setrons”) that is well established for clinical use, especially because of their effects on Chemotherapy-Induced Nausea and Vomiting (CINV). Among them, [**^18^F]Fesetron** was designed based on the model of **[^125^I]MIZAC**, a potent and selective radioligand of 5-HT_3_R [175]. **[^18^F]Fesetron** was shown to display a binding profile to rat brain regions known to contain a high density of 5-HT_3_R. However, the in vivo rat brain uptake of **[^18^F]Fesetron** was low, and a significant amount of activity was unable to cross the BBB. This lack of brain permeability, which could be explained by low lipophilicity, limits the usefulness of **[^18^F]Fesetron** as a PET radiotracer in this animal model [177].

The second “setron” drug developed in 2016 was a fluorine analogue of the clinically used **palonosetron** which also contained the 3-aminoquinuclidinyl moiety. In vitro autoradiography showed specific labeling in rodent brain slices, but in vivo PET studies in rats with **[^18^F]Fluoropalonosetron** gave low and homogeneous radiotracer signals [178]. This could be related to the low expression of 5-HT_3_R in the brain combined with the low lipophilicity of the radioligand and could explain why **[^18^F]Fluoropalonosetron** is not suitable for in vivo PET imaging of 5-HT_3_ receptors.

### 4.4. Miscellaneous Derivatives as 5-HT_3_R Radiotracers

**Figure 13:**
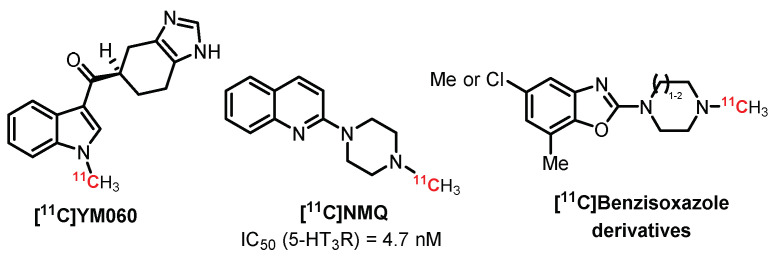
Miscellaneous derivatives as 5-HT_3_R radiotracers.

In 1995, **[^11^C]YM060** was labeled with carbon-11 through *N*-methylation of indole and revealed poor brain uptake in mice (Figure 13) [179]. *N*-methylquipazine (**NMQ**, (2-[1-(4-methyl)-piperazinyl]quinoline) is an agonist with good affinity and selectivity for 5-HT_3_R (IC_50_ = 4.7 nM). Its biodistribution has been evaluated in vivo in rats and nonhuman primates using PET imaging [180]. The imaging results indicate that its uptake was observed in structures known to contain 5-HT_3_R, although the radiotracer also interacted with non-5-HT_3_R sites. In 2008, Gao et al. designed a new carbon-11-labeled benzisoxazole derivative for PET imaging of 5-HT_3_ receptors [181]. These partial agonists exhibited high binding affinity and could be promising for future in vivo biological evaluation.

## 5. 5-HT_4_ Receptors

5-HT_4_R receptors were first described by Dumuis et al. in 1988 [182]. They are GPCRs positively coupled to adenylyl cyclase and induce cyclic adenosine monophosphate (cAMP) production [183]. 5-HT_4_Rs are located in the peripheral nervous system (PNS), mainly being found in the heart, gastrointestinal tract, and enteric nervous system [184], as well as in the CNS, where they are highly expressed in different brain regions like the hippocampus, amygdala, and cerebral cortex, suggesting the involvement of the receptor in brain physiological functions [185] such as learning and memory [186,187,188], eating disorders [189], and mood behavior [190]. In order to further specify in vivo the 5-HT_4_R physiological or pathological role, selective radioligands able to target 5-HT_4_R have been developed for SPECT and PET applications.

### 5.1. Benzodioxane Series: **SB207710** Derivatives

**Figure 14:**
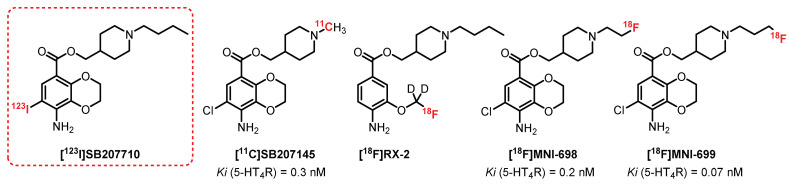
**SB207710** derivatives as 5-HT_4_R radioligands.

Kaumann et al. [191] reported on **SB207710**, a selective high-affinity benzodioxane antagonist for 5-HT_4_R (Figure 14). Autoradiographic experiments on humans [192] and on rat brains with its radiolabeled analogue, **[^125^I]SB207710,** confirmed its potency and selectivity [193] and led Pike et al. to characterize its efficacy in vivo as a SPECT radioligand [194]. A good brain uptake in rats was observed for **[^125^I]SB207710**, and the study revealed specific radiolabeling of regions rich in 5-HT_4_R (striatum, frontal, and temporal cortex). **[^123^I]SB207710** was then examined by SPECT imaging in nonhuman primates. The radiotracer readily accumulated in 5-HT_4_R-rich areas. The specificity of this radiolabeling was underlined through pretreatment with a selective 5-HT_4_R antagonist, **SB204070,** which reduced the radioactivity in all brain regions. Even if **[^123^I]SB207710** entered the brain, its signal quickly disappeared in SPECT, thus proving rapid clearance. Since then, no further investigations for SPECT studies in humans have been reported. Nevertheless, the use of **[^123^I]SB207710** in SPECT imaging provided the first demonstration of 5-HT_4_R imaging in primate brains in vivo [194]. The antagonist **SB207145** (Ki = 0.3 nM), structurally related to **SB207710**, was radiolabeled with carbon-11 by Gee et al. in 2008 [195], and its potential as a PET radioligand was evaluated for 5-HT_4_R imaging. The preliminary data in a few healthy subjects indicated that **[^11^C]SB207145** was distributed in the brain according to the known 5-HT_4_R distribution. It was then successfully used for in vivo studies in animals to evaluate the metabolism and binding kinetics in a minipig brain [196] and human subjects [195,197,198,199]. To date, **[^11^C]SB207145** remains the only 5-HT_4_R radiotracer that has been evaluated in human studies [200,201,202,203]. However, the short half-life of the radioisotope (t_1/2_ = 20.4 min) significantly restricted its use for more advanced clinical trials.

In 2010, Xu et al. reported the first fluorine-18-radiolabeled 5-HT_4_R radiotracer **[^18^F]RX-2** [204] in which deuterium was incorporated to provide greater in vivo resistance to defluorination [205]. PET studies with **[^18^F]RX-2** were performed on monkeys in 2014 and showed a high brain uptake, quantifiable distribution, and insignificant radiometabolite detection [206]. The mild but stable 5-HT_4_R-specific signal validated the potential use of **[^18^F]RX-2** for mapping 5-HT_4_R, but no transposition to human PET studies were published to date.

More recently, **[^18^F]MNI-698** and **[^18^F]MNI-699**, two fluorinated analogues of **SB207145** containing fluorine-18 in the terminal piperidine alkyl chain, were described [207]. These radioligands have been shown to be affine with 5-HT_4_R (Ki = 0.2 nM and 0.07 nM, respectively). In vivo PET studies on adult rhesus monkeys established that both radioligands displayed suitable brain penetration and brain distribution consistent with the known 5HT_4_R densities. The whole-body biodistribution and dosimetry of **[^18^F]MNI-698** were then investigated and supported that this radioligand could be used for investigation in humans [208,209]. All these ligands belong to the same family of compounds and contain an ester function which can generate short metabolic stability. Other chemical series deprived of ester functions have been explored recently, such as azaphenanthrene derivatives [210].

### 5.2. 5-HT_5_ Receptors

Two subtypes of 5-HT_5_R are known—5-HT_5A_R and 5-HT_5B_R—but only 5-HT_5A_R is expressed in the human brain. Because of the lack of a selective and potent ligand described for this receptor to date, no potent radiotracer or even radioligand has been described.

### 5.3. 5-HT_6_ Receptors

The 5-HT_6_ receptor is one of the most recently identified members of the 5-HT receptor family. First cloned in a rat, the 5-HT_6_R gene encodes a 438-aminoacid protein [211,212] which is well-conserved in humans [213], and 5-HT_6_R is a G_αs_-coupled receptor (GPCR) that is almost exclusively expressed in the CNS and is abundant in the brain regions involved in cognitive functions, such as the prefrontal cortex, hippocampus, striatum, and nucleus accubens [214,215,216]. Similar distribution patterns between rats, nonhuman primates, and humans have been established [217,218]. In addition to the adenylyl cyclase signaling pathway [219], 5-HT_6_R has been linked to cellular signaling cascades involved in cognitive processes and neurogenesis, such as the mammalian target of the rapamycin (mTOR) pathway [220]. Thus, 5-HT_6_R has emerged as a promising target for cognitive enhancement in neurodegenerative or in psychiatric diseases and for antiobesity drugs [187,221,222,223,224,225].

### 5.4. Benzene Sulfonamide Derivatives: **SB258585** Derivatives

**Figure 15:**
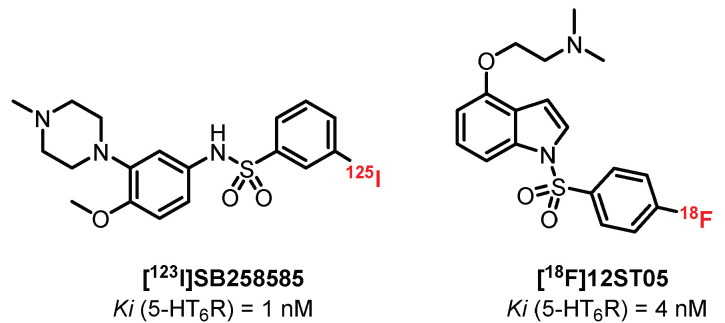
**[^125^I]SB258585** and **[^18^F]12ST05**.

Selective radioligands for in vitro autoradiographic studies are available, such as **[^125^I]SB258585** (Ki = 1 nM), but their development as in vivo imaging tools was restricted due to poor brain uptake (Figure 15) [226]. Tang et al. [227] developed a series of 4-(2-aminoethoxy)-*N*-(phenylsulfonyl)indoles based on the selective 5-HT_6_R antagonist **SB271046** [228]. This study led to the design of **[^18^F]12ST05**, presenting good affinity for 5-HT_6_R (Ki = 4 nM). Ex vivo autoradiographic studies in rat brain slices established high labeling in regions known to contain 5-HT_6_R. In vivo studies in cats failed to show the specific binding of **[^18^F]12ST05** to 5-HT_6_R in the cerebral regions, and hence, further investigations were not undertaken for **[^18^F]12ST05** as a PET radioligand.

### 5.5. 5-(Piperazin-1-yl)Quinolone Derivatives: **GSK215083** Derivatives

**Figure 16:**
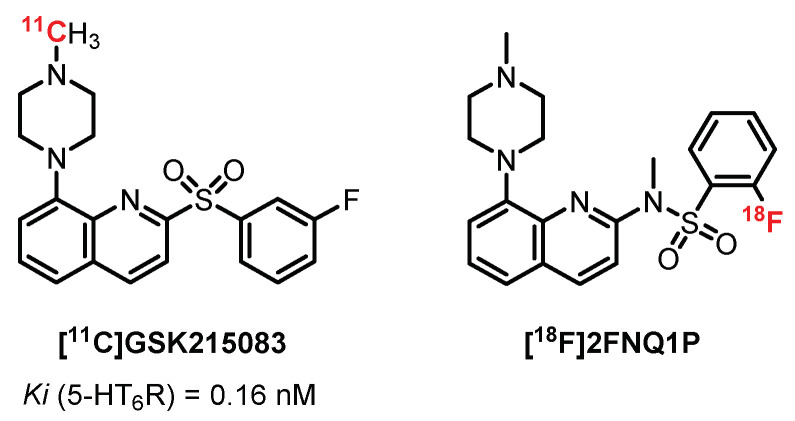
**[^11^C]GSK215083** and **[^18^F]2FNQ1P**.

In 2012, Parker et al. published an interesting study presenting the in vivo evaluation of **[^11^C]GSK215083** (Ki (5-HT_6_R) = 0.16 nM and Ki (5-HT_2A_R) = 0.79 nM) in pigs, nonhuman primates, and humans (4 healty volunteers) (Figure 16). They revealed a significant binding of the radiotracer on the striatum and frontal cortex in correlation with the autoradiograms carried out previously on cats. Two additional studies confirmed the interest shown in this radiotracer and revealed a decrease in 5-HT_6_R with age. Moreover, its mixed action (5-HT_6_R and 5-HT_2A_R) was effective in the study of neuropsychiatric diseases such as AD and schizophrenia [229,230].

In 2014, Rosse designed radiolabeled quinolone derivatives structurally related to **GSK215083** as 5-HT_6_R PET ligands [231]. In 2014, Colomb et al. developed a new chemical series of compounds inspired by **GSK215083** in order to maintain a strong affinity toward 5-HT_6_R and to optimize its specificity. The bioisosteric replacement of the sulfonyl part by sulfonamide led to a new ligand, **2FNQ1P**, with high affinity and selectivity for 5-HT_6_ receptors [232]. The distribution of **[^18^F]2FNQ1P** in 5-HT_6_R-dense regions (i.e., the cortex and striatum) was assessed by semiquantitative autoradiography in rat brains. Furthermore, the addition of **SB2258585** (a specific 5-HT_6_R antagonist) displaced the binding of **[^18^F]2FNQ1P** in a concentration-dependent manner, confirming its excellent specificity for 5-HT_6_R [233]. The cerebral distribution of **[^18^F]2FNQ1P** was then studied in vivo in various animal models, from rodents to nonhuman primates. The in vivo evaluation in rats using microPET exhibited reduced brain uptake of **[^18^F]2FNQ1P**. By contrast, in vivo PET imaging with **[^18^F]2FNQ1P** in feline and primate models showed high uptake and distribution in the striatal regions particularly rich in 5-HT_6_R, suggesting that **[^18^F]2FNQ1P** could be a suitable 5-HT_6_R PET tracer for quantification of the brain 5-HT_6_R in primates. A recent in vitro study demonstrates a decrease in the caudate nucleus 5-HT_6_R density with **[^18^F]2FNQ1P** in human brain tissue from patients with different stages of Alzheimer’s disease [233]. Future in vivo exploration should be carried out to establish the relevance of **[^18^F]2FNQ1P** to confirm an early decline in 5-HT_6_R expression during the progression of the disease.

### 5.6. 5-HT_7_ Receptors

In 1993, 5-HT_7_R was independently cloned and discovered by several scientific teams in different species including mice, rats, and humans [212,234,235]. In human, the protein is encoded by the HTR_7_ gene located on the long arm of chromosome 10 (10q23.31), comprising 3 introns involved in alternative splicing phenomena. Splicing of intron 2 and 3 is responsible for the existence of 3 distinct isoforms—5-HT_7A_, 5-HT_7B_ and 5-HT_7D_—showing no pharmacological difference. The 5-HT_7_R receptors are GPCRs (coupled to a Gs protein) that will diffuse nerve impulses through the activation of adenylate cyclase and the production of the secondary messenger: cAMP. In human CNS, 5-HT_7_Rs are localized mainly in the anterior thalamus and in the dentate gyrus. The hypothalamus, anterior cingulate gyrus, hippocampus, and amygdala constitute other human brain regions containing substantial amounts of 5-HT_7_Rs [236].

The 5-HT_7_R receptors are involved in the phenomena of learning and memorization as well as in the regulation of temperature and circadian rhythm. In addition, they play a decisive role in the development of certain pathologies such as schizophrenia, migraines, and Alzheimer’s disease [237]. Among the 14 known serotonergic receptors, 5-HT_7_R is the most recently discovered and explored one. Its implication in the genesis of certain pathologies such as depression, anxiety, and epilepsy make it a target of choice for the development of curative and diagnostic tools [238]. The development of radiotracers allowing their visualization and quantification has become a major axis of research in medicinal chemistry.

### 5.7. Oxindoles Series: **DR4004** Derivatives

**Figure 17:**
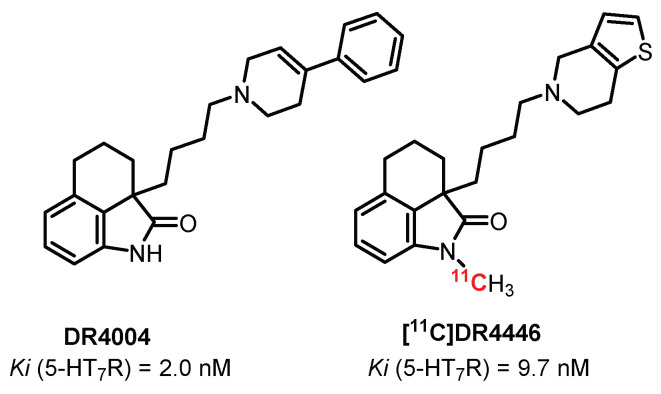
**DR4004** and **[^11^C]DR4446**.

**DR4004** (Figure 17) is a selective and potent antagonist of 5-HT_7_ receptors (Ki = 2 nM) discovered in 1999 by Kikuchi et al. [239]. In 2002, the same group proposed a N-methylated analogue, **DR4446**, while maintaining the affinity and selectivity toward 5-HT_7_R (Ki = 9.7 nM) [240]. Radiolabeling through [^11^C]N-methylation, performed by Zhang et al., allowed obtaining the first imaging study with the 5-HT_7_R antagonist radiotracer [241]. **[^11^C]DR4446** showed good diffusion through the BBB in vivo (rhesus monkeys), with a high brain uptake especially in the 5-HT_7_R-rich region: the thalamus. However, this study did not allow a favorable transfer to human experiments due to the impossibility to observe a blocking effect in a self-block study, probably due to off-target labeling of other CNS receptors.

**Figure 18:**
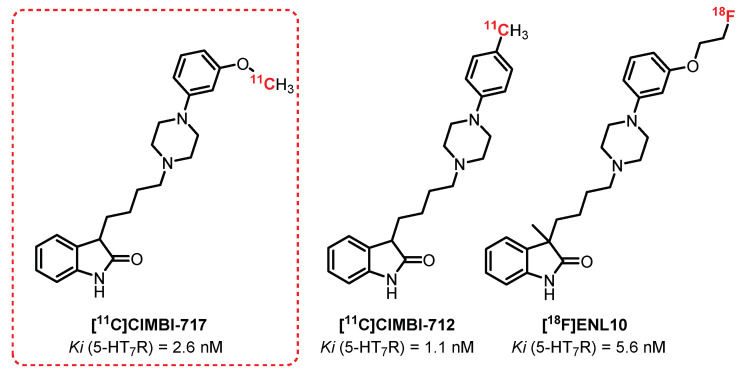
**[^11^C]CIMBI-717**, **[^11^C]CIMBI-712**, and **[^18^F]ENL10**.

**CIMBI-717** and **CIMBI-712** (Figure 18) are potent 5-HT_7_R antagonists (Ki = 2.6 nM and 1.1 nM, respectively) discovered in 2012 by Herth et al. [242]. In 2014, both of these ligands, after radiolabeling with carbon-11, were evaluated in vivo (pigs) as 5-HT_7_R tracers by Knudsen et al. A high brain uptake was observed for both **[^11^C]CIMBI-712** and **[^11^C]CIMBI-717.** Their specificity was demonstrated through blocking studies after the injection of **SB-269970,** a 5-HT_7_R antagonist [243]. For **[^11^C]CIMBI-717**, a radiolabeling of the thalamus, the striatum, the hippocampus, and the cortex in correlation with the in vitro autoradiographs determined beforehand with **[^3^H]SB-269970** was also observed. Despite these encouraging results, no recent publication mentions its use in humans. However, in the same series, Herth et al. proposed in 2019 a closely related analogue of **CIMBI-717** and **CIMBI-712**: **ENL10** [244] while overcoming the problematic short half-life of carbon-11 through fluorine-18 labeling. **[^18^F]ENL10**, a potent and selective 5-HT_7_R antagonist (Ki = 5.6 nM) demonstrated in vivo (rats) a very modest passage across the BBB due to the efflux phenomena induced by a significant expression of the transporter P-gp in rats. In the presence of **Elacridar** (P-gp inhibitor) and **SB-269970**, the study revealed specific radiolabeling of 5-HT_7_R-rich areas, making this compound a serious candidate for possible application of these tests to other species (e.g., pigs or nonhuman primates). This suggestion is motivated mainly by recent observations, indicating a lower expression of P-gp in non-rodents and making the use of **Elacridar** probably obsolete for these species.

### 5.8. N-Sulfopyrrolidine Series: **SB-269970** Derivatives

**Figure 19:**
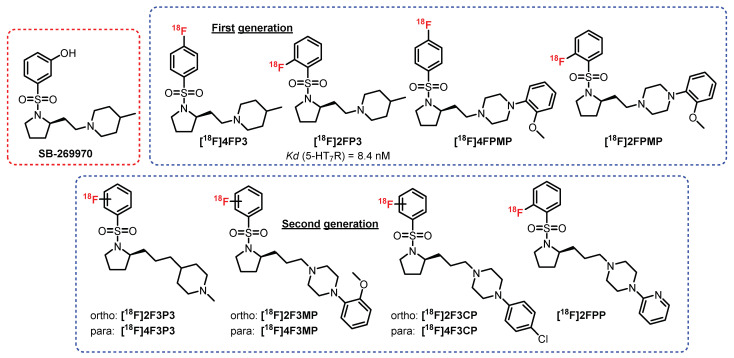
Radiolabeled **SB-269970** derivatives.

In 2011, Zimmer et al. developed a new chemical series of compounds inspired by the *N*-sulfopyrrolidine structure from a 5-HT_7_R antagonist: **SB-269970** [243] (Figure 19). Among this first generation of radioligands, **[^18^F]2FP3** proved to be the most promising. Indeed, **[^18^F]4FP3**, **[^18^F]4FPMP**, and **[^18^F]2FPMP** exhibited nonspecific radiolabeling in the presence of a reference antagonist (**SB269970**) in rats (ex vivo and in vivo). **[^18^F]2FP3** is an affine and selective 5-HT_7_R antagonist (K_D_ = 8.4 nM) which was previously tested in rats (ex vivo autoradiography) and in cats (in vivo) [243]. The first results were very promising, since selective radiolabeling of the hippocampus, cingulate cortex, and thalamus was observed. In addition, 95% of the collected signal was assigned to **[^18^F]2FP3** and not from a radiometabolite. In 2019, a complementary study on pigs and nonhuman primates was carried out [245]. Partial specificity of **[^18^F]2FP3** for the cerebellum (in monkeys) was revealed, clearly reflecting the difficulties in translating the results from one species to another. In addition, **[^18^F]2FP3** was found to be rapidly metabolized in pigs (50% metabolized 10 min after injection) and more moderately in primates (50% in 60 min). This study did not provide a favorable conclusion for its use in humans. This first approach was further completed by the evaluation of a second generation of radioligands, where additional carbon between the pyrrole and the piperidine or the piperazine was inserted [246]. These radioligands were optimized in terms of radiolabeling specificity for 5-HT_7_R, but additional pharmacomodulations will be necessary in order to improve the biodistribution and passage of the BBB.

**Figure 20:**
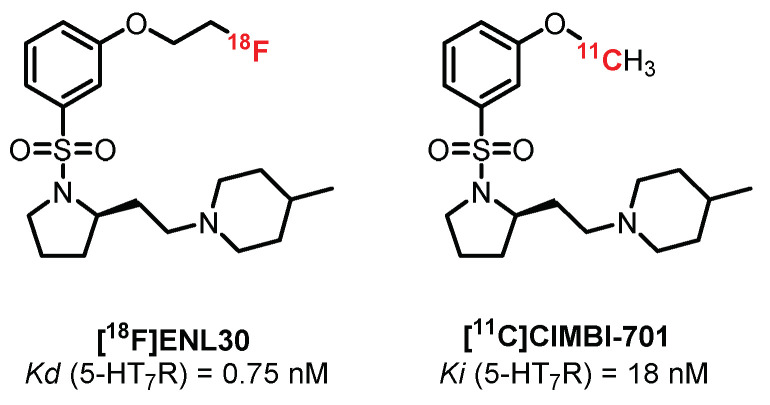
**[^18^F]ENL30** and **[^11^C]CIMBI-701**.

Still in the *N*-sulfopyrrolidine series, two radioligands were synthesized and tested by Herth et al.: **[^18^F]ENL30** [247] and **[^11^C]CIMBI-701** [248] (Figure 20). These compounds exhibited mixed labeling of the 5-HT_7_R and σ-receptors and added to a moderate signal in the brain (probably linked to the mechanisms of efflux (P-gp substrate)), thus limiting their use in humans.

## 6. Serotonin Transporter

A serotonin transporter (SERT) is not a serotonergic receptor per se. However, it is heavily involved in the regulation of 5-HT. The development of radioligands that are affine and selective for these transporters constitutes an interesting axis of development in clinical research.

SERTs are proteins consisting of 12 transmembrane helices whose main role is to regulate the concentration of 5-HT in the inter-synaptic space. The reuptake of 5-HT results from a complex catalytic reaction incorporating both symporter and antiporter transport involving three distinct ions: Na^+^, Cl^−^, and K^+^. This transport takes place in four successive stages [249]. (1) A conformation change of SERTs, resulting in exposure of the binding sites to the intracellular medium, takes place due to the extracellular stoichiometric binding of 5-HT, Na^+^ ions, and Cl^−^ ions. (2) The 5-HT, Na^+^, and Cl^-^ ions are released in the intracellular medium. (3) Intracellular binding of K^+^ ions leads to a conformational modification of the SERT, which regains its original conformation. (4) K^+^ ions are released into the extracellular medium. SERT expression is tightly regulated by the concentration of 5-HT in the intersynaptic space. In the presence of an increased amount of 5-HT, there is a decrease in the internalization of the transporter, resulting in an increase in its density on the presynaptic membrane. On the contrary, SERTs are downregulated in the presence of a small amount of 5-HT. In this context, a large panel of compounds has been developed with the main objective of blocking the reuptake of serotonin by inhibiting these transporters. The serotonin reuptake inhibitors (SSRI drug class), including **fluoxetine** (Prozac^®^), **sertraline** (Zoloft^®^), and **citalopram** (Celexa^®^), are mainly prescribed to treat major depressive disorders as well as obsessive-compulsive disorders.

During the last three decades, the development of new potent and selective radiotracers of SERTs has become a major axis of development in clinical and preclinical research. Indeed, their expression fluctuations, dependent on the endogenous 5-HT level, are an interesting marker in various pathologies such as depression, bipolarity, and eating disorders. In addition, these studies could allow assessing the impact of regular administration of SSRIs on the serotonergic system, thus allowing a better understanding of their mode of action.

### 6.1. Isoquinoline Series: **McN5652** Derivatives

**Figure 21:**
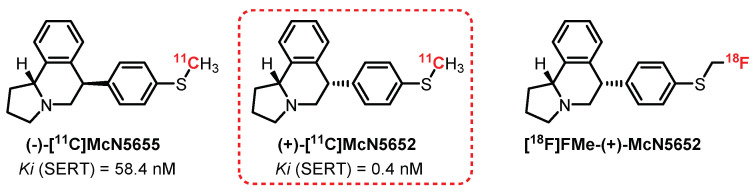
**(−)-[^11^C]McN5655**, **(+)-[^11^C]McN5652**, and **[^18^F]FMe-(+)-McN5652**.

**McN5652** is the first SERT ligand evaluated as a radiotracer. The two evaluated diastereomers of **McN5652**—**(+)-[^11^C]McN5652** and **(−)-[^11^C]McN5655**—present significant differences in terms of affinity toward SERTs at Ki of 0.4 nM and 58.4 nM, respectively (Figure 21) [250]. Preliminary studies carried out on mice allowed appreciating the difference in radiolabeling between the two enantiomers, with clearly more interesting results for the (+) enantiomer. In 1995, a study on baboon brains revealed significant absorption of the radiotracer in the midbrain, hypothalamus, thalamus, and striatum. This distribution correlates with the results obtained for brain section autoradiography with **[^3^H]paroxetine** [251]. In 2000, Parsey et al. carried out a study on six healthy male volunteers and confirmed the interest in **(+)-[^11^C]McN5652** as a radiotracer, allowing the quantification of SERTs in the limbic, striatal, and thalamic regions. However, the study revealed major limitations to its use, in particular with the presence of a non-selective labeling in the neocortex area and a specific or nonspecific binding ratio (satisfactory after 115 min) not being in adequacy with the use of carbon-11 (t_1/2_ = 20.4 min) [251,252]. Nevertheless, **(+)-[^11^C]McN5652** has been used on numerous occasions to assess SERT biodistribution in various pathological conditions such as depression and obsessive-compulsive and mood disorders [253,254,255].

In order to improve its properties, a methylfluorinated analogue of **(+)-[^11^C]McN5652** was synthesized: **[^18^F]FMe-(+)-McN5652**. Preliminary evaluations in a rat (ex vivo) revealed intense and specific radiolabeling (competitive tests in the presence of **fluoxetine**, **nisoxetine**, and **GBR12909**) of the amygdala, hypothalamus, raphe nuclei, thalamus, and locus coeruleus compared with the cerebellum (nonspecific binding) [256]. A complementary study on a pig (in vivo) allowed concluding favorably regarding the use of this radiotracer as an alternative to **(+)-[^11^C]McN5652** [257]. However, as its affinity for SERTs was found to be more moderate (Ki = 2.3 nM) than **(+)-[^11^C]McN5652**, its binding parameters were estimated to be less reliable [258]. Despite promising results, no publication to date mentions the use of this tracer in humans.

### 6.2. Diarylthioether Series: **[^123^I]IDAM** Derivatives

**Figure 22:**
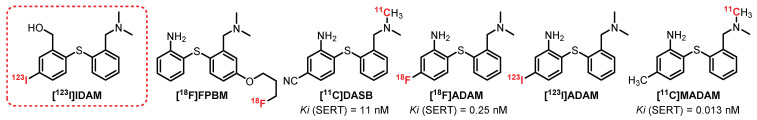
**[^123^I]IDAM** derivatives.

**[^11^C]DASB** is probably the most used radiotracer to explore the distribution of SERTs at the CNS level. This radiotracer was synthesized in the early 2000s as an analogue of **[^123^I]IDAM,** the first radiotracer to be synthesized and evaluated in the diarylthioether series [259], but quickly abandoned in favor of its analogues (Figure 22). **[^11^C]DASB** is very affine and selective toward SERT (Ki = 1.1 nM) [260]. The first human study (nine healthy volunteers) was performed by Wilson et al. and allowed observing an intense assimilation of **[^11^C]DASB** in the midbrain, the thalamus, the hypothalamus, and the striatum [261]. The specificity was confirmed by a prior oral intake of **citalopram**, decreasing the radioactivity of these cerebral areas to equivalent values to those obtained in the cerebellum (nonspecific labeling). In addition, comparative tests with **(+)-[^11^C]McN5652** indicated a much more interesting specific or nonspecific binding ratio for **[^11^C]DASB** [262,263]. Between 2002 and 2004, several studies were carried out on animals (rats, cats, and baboons), allowing for evaluating the general distribution of **[^11^C]DASB**, and concluded favorably regarding its use to determine the influence of SERTs in various psychiatric and neurological disorders [264,265,266]. Thus, **[^11^C]DASB** was used on numerous occasions in patients presenting various pathologies such as depression, schizophrenia, obsessive-compulsive disorder, Parkinson’s disease, alcoholism, and bipolarity [267,268,269,270,271,272,273]. To date, **[^11^C]DASB** constitutes a reference in the quantification and characterization of SERTs at the cerebral level.

In 2001, **[^11^C]MADAM**, a methylated analogue of **[^11^C]DASB** exhibiting a very strong affinity and specificity for SERTs (Ki = 0.013 nM), was described [274]. The first baboon studies indicated consistent radiolabeling of SERT-rich areas of the brain [275]. In 2005, imaging studies on healthy humans (nine volunteers) confirmed the previous observations, with a major absorption of **[^11^C]MADAM** in the raphe nuclei, the putamen, the hippocampus, and more moderately in the frontal and cingulate cortex [276,277]. Finally, **[^11^C]MADAM** has been used more recently to study the density of brain SERTs in people with eating disorders and gambling addiction [278].

In 2000, the discovery of **[^123^I]ADAM** offered the possibility to perform human imaging exploration of SERTs using SPECT imaging [279]. **[^123^I]ADAM** was first tested in mice, rats, rabbits, and nonhuman primates and allowed observing intense and specific radiolabeling of the midbrain [280,281,282,283]. In 2003 and 2004, two preliminary studies evaluated the biodistribution of **[^123^I]ADAM** in humans (7 and 11 healthy volunteers) and allowed observing the specific labeling of the midbrain [284,285]. These results were confirmed by several additional studies which demonstrated a specific and reproducible labeling in the midbrain, the thalamus, and the striatum, particularly in people with an SERT polymorphism [286,287,288]. Finally, a double-blind trial was carried out (12 healthy men volunteers) in order to visualize the differences in radiolabeling with an administration of **paroxetine** versus a placebo [289]. **[^123^I]ADAM** allows exploration of the variations of SERTs, particularly in the presence of various pathologies such as depression, Parkinson’s disease, and eating disorders [289,290,291,292,293,294]. In 2003, **[^18^F]ADAM**, a fluorinated analogue of **[^123^I]ADAM** with good affinity and selectivity toward SERTs (Ki = 4.8 nM), was described [295,296]. **[^18^F]ADAM** was first tested on monkeys and allowed observing an intense radiolabeling of the striatum, thalamus, and midbrain. The selectivity was confirmed in the presence of increasing doses of **fluoxetine**, allowing visualization of the displacement of the radioligand [297]. **[^18^F]ADAM** was then further studied in humans with major depressive disorders and allowed the observation of a significant decrease in radiolabeling in the midbrain and the striatum in those who had attempted suicide recently. These contradictory results are probably linked to a recent administration of an SSRI, which can induce a decrease in the central SERTs [298,299]. The results obtained with **[^18^F]ADAM** are very encouraging and place it as a future radiotracer for exploring and quantifying the central SERTs using PET. A closely related analogue of **[^18^F]ADAM** was described in 2009 by Wang et al., exhibiting high affinity and selectivity toward SERTs (Ki = 0.25 nM): **[^18^F]FPBM** [300]. The first studies in animals (rats) revealed specific labeling of the midbrain, cortex, striatum, and thalamus. **[^18^F]FPBM** evaluation was recently supplemented by tests on monkeys, which confirmed the interest of this compound as a new PET tracer of central SERTs [301].

### 6.3. Tropane Series: **β-[^123^I]CIT** Derivatives

**Figure 23:**
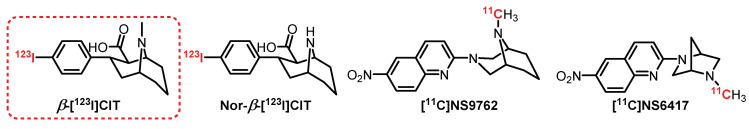
SERT tracer in the tropane series.

***β*-[^123^I]CIT** (Figure 23) is one of the first radiotracers to be synthesized and tested for SPECT imaging in humans [302,303,304]. ***β*-[^123^I]CIT** has a mixed affinity for serotoninergic and dopaminergic transporters, which is not a major drawback since there is a different distribution within the brain for these two targets. However, its demethylated counterpart **Nor-*β*-[^123^I]CIT** has an affinity 10 times higher for SERTs and has allowed the observation in humans of a very strong accumulation of radioactivity in the midbrain and the striatum [305]. Since then, ***β*-[^123^I]CIT** and **Nor-*β*-[^123^I]CIT** have been used repeatedly to determine the evolution of the SERT density in the presence of pathologies such as depression and autism and in people with obsessive-compulsive disorders [306,307,308]. Recently, compounds exhibiting similar structures have been synthesized, particularly **[^11^C]NS9762** and **[^11^C]NS6417**, which could provide promising alternatives in the coming years [309].

## 7. Conclusions

As described in this review, several potent and selective radiotracers for 5-HTR and SERTs have been developed in recent decades. Three radiotracers with good affinity to 5-HT_1A_R are commonly used in clinical trials: **[^11^C]WAY100635**, **[^18^F]MPPF**, and **[^18^F]FCWAY**. The characterization and distribution of 5-HT_2A_R is frequently performed by **[^123^I]5-I-R91150** in SPECT imaging, with that of **[^18^F]Setoperone**, **[^18^F]Altanserine**, and **[^11^C]MDL100907** performed in PET imaging. Moreover, the utilization of **[^123^I]ADAM**, **[^11^C]DASB**, and **[^11^C]MADAM** has improved the exploration of SERTs. These radiotracers have greatly contributed to facilitating the characterization, localization, and biological implication of the brain serotonin system in normal and pathological conditions. Indeed, in vivo PET and SPECT studies carried out on humans and animals allowed the observation of brain areas rich in 5-HTR by precise detection of radioactive emission. The signal specificities were confirmed via radiotracer displacement methods in the presence of reference ligands. Furthermore, these studies have shown the importance of multiparametric approaches to designing a new radiotracer, since a lack of selectivity, insufficient passage through the BBB, and fast metabolization can significantly decrease its effectiveness. In addition, these investigations also revealed the difficult transposition of the results from one species to another due to the interspecies variation of 5-HTR distribution, which could lead to misinterpretation. Overall, despite interesting results during clinical trials, none of these radiotracers are approved as diagnostic tools for neuropsychiatric disorders in clinics. New radiolabel-capable, potent, and selective ligands of the 5-HTRs allowing exploration of brain functions are thus still needed to fully understand the role of these receptors in either healthy or sick individuals.

## Data Availability

Data sharing not applicable.
